# Lean Psoas Muscle Area Is Associated with Length of Stay After Lower Limb Revascularization for CLTI

**DOI:** 10.3390/diagnostics16111621

**Published:** 2026-05-26

**Authors:** Jagoda Bobula, Joanna Halman, Kamil Myszczyński, Jakub Dybcio, Nina Kimilu, Agnieszka Blacha, Grzegorz Owedyk, Mariusz Siemiński

**Affiliations:** 1 Student Scientific Circle of Vascular Surgery, Faculty of Medicine, Medical University of Gdansk, 80-210 Gdansk, Poland; 2 Vascular Surgery Department, Medical University of Gdansk, University Clinical Centre in Gdansk, 80-952 Gdansk, Poland; joannahalman@gmail.com; 3 Centre of Biostatistics and Bioinformatics Analysis, Medical University of Gdansk, 80-210 Gdansk, Poland; 4 Department of Clinical Nutrition and Dietetics, Medical University of Gdansk, 80-210 Gdansk, Poland; 5 Scientific Circle of Neurotraumatology, Department of Emergency Medicine, Medical University of Gdansk, 80-210 Gdansk, Poland; 6 Department of Emergency Medicine, Medical University of Gdansk, University Clinical Centre in Gdansk, 80-952 Gdansk, Poland; mariusz.sieminski@gumed.edu.pl

**Keywords:** chronic limb-threatening ischemia, revascularization, length of stay, predictors, LPMA, sarcopenia

## Abstract

**Background**: Chronic limb-threatening ischemia (CLTI) is associated with high morbidity and substantial healthcare utilization. Length of hospital stay (LOS) after lower limb revascularization is influenced by procedural complexity, but patient physiological reserve may also play a role. We evaluated whether CT-derived lean psoas muscle area (LPMA) is independently associated with LOS in patients undergoing revascularization for CLTI. **Methods**: We retrospectively analyzed 234 consecutive patients treated with endovascular, hybrid, or open revascularization for CLTI (Rutherford 4–5) between 2018 and 2021. Sarcopenia markers were derived from preoperative CT at the L3 level, including psoas muscle area (PMA), muscle density (PMD), and LPMA. Multivariable linear regression models with log-transformed LOS were used to estimate relative effects on hospitalization duration. **Results**: Median age was 68 years and 65.4% were male; 76.5% of admissions were urgent. Median LOS was 6 days (IQR 4–9). Procedure type was the strongest determinant of LOS: hybrid (β = 0.69, *p* < 0.001) and open surgery (β = 0.73, *p* < 0.001) were associated with approximately 99% and 108% longer LOS compared with endovascular treatment. Higher LPMA was independently associated with shorter LOS (β = −0.00049, *p* = 0.004). Smoking (β = −0.21, *p* = 0.003) and history of myocardial infarction (β = −0.19, *p* = 0.030) were associated with shorter LOS, whereas dialysis showed a non-significant trend toward longer hospitalization (β = 0.36, *p* = 0.056). **Conclusions**: In patients undergoing lower limb revascularization for CLTI, CT-derived LPMA demonstrated a modest but independent association with hospital stay duration after adjustment for procedural and clinical factors. Given the exploratory nature of this study, these hypothesis-generating findings support further evaluation of imaging-based muscle assessment as an adjunct marker of physiological reserve in this high-risk population.

## 1. Introduction

Chronic limb-threatening ischemia (CLTI) represents the most advanced stage of peripheral arterial disease and is associated with high morbidity, mortality, and healthcare costs. Limb salvage frequently requires urgent revascularization; however, postoperative recovery is influenced not only by the procedure itself but also by the patient’s underlying physiological reserve. Prolonged hospital length of stay (LOS) after lower limb revascularization has important consequences, including increased risk of hospital-acquired complications, greater resource utilization, and delayed return to baseline function.

While previous studies have primarily focused on procedural characteristics and traditional risk factors such as age, comorbidities, and operative urgency, growing evidence suggests that global health status and nutritional reserve independently affect postoperative outcomes [1,2,3,4]. Imaging-based markers of sarcopenia offer a rapid and objective assessment of skeletal muscle status using routinely acquired preoperative computed tomography. The lean psoas muscle area (LPMA), which integrates muscle area and density, reflects both muscle quantity and quality and may therefore provide a clinically meaningful estimate of physiological reserve.

Importantly, these data are available before surgery, allowing early identification of patients who may benefit from targeted perioperative strategies such as prehabilitation, nutritional optimization, and early mobilization. Such interventions are not yet systematically implemented in CLTI management, where the limited time between diagnosis and revascularization often restricts preoperative optimization. Integrating sarcopenia assessment into routine preoperative evaluation could support more individualized care aimed at reducing LOS and improving recovery [5,6].

The primary objective of this study was to evaluate whether CT-derived sarcopenia markers, like LPMA, are independently associated with LOS in patients undergoing revascularization for CLTI.

## 2. Materials and Methods

### 2.1. Study Design and Population

The study was conducted as a retrospective observational study of 234 consecutive patients undergoing lower limb revascularization for CLTI at the Vascular Surgery Department, University Clinical Center, Gdansk, Poland, between 1 March 2018 and 31 December 2021. Open, hybrid, and endovascular procedures were included. Inclusion criteria comprised patients aged ≥18 years who underwent primary revascularization for chronic limb-threatening ischemia (Rutherford categories 4–5) and had preoperative computed tomography angiography (CTA) including the L3 vertebral level performed within 12 months prior to surgery. Exclusion criteria were emergency surgery for acute limb ischemia, absence of preoperative CTA in our center’s records, incomplete clinical data, or prior major amputation of the index limb. The study was conducted in accordance with the Declaration of Helsinki and approved by the Independent Bioethical Committee of the Medical University of Gdansk (protocol code KB/340/2024; approval date: 5 July 2024).

### 2.2. Data Collection

Demographic characteristics, comorbidities, procedural details (open, hybrid, or endovascular), and postoperative outcomes were extracted from the institutional vascular registry and electronic medical records. Imaging-based sarcopenia markers were derived from preoperative computed tomography scans at the L3 vertebral level. The primary outcome of the study was length of hospital stay (LOS) following lower limb revascularization for CLTI. Length of hospital stay (LOS) was defined as the total duration from the day of hospital admission to the day of formal discharge (total admission-to-discharge duration). This total timeframe was utilized to fully capture the preoperative optimization period (e.g., infection control, volume resuscitation, and stabilization of comorbidities), which represents an integral part of the overall hospitalization burden in patients with CLTI.

#### 2.2.1. Cohort Selection and Excluded Cases

Of 397 consecutive patients undergoing lower limb revascularization for CLTI during the study period, 163 were excluded due to absence of preoperative CTA including the L3 vertebral level. Compared with the analyzed cohort, excluded patients were more frequently elective admissions and had shorter LOS (4.5 vs. 6 days, *p* < 0.001). A detailed comparison of included and excluded patients is presented in Supplementary Table S1. Patients classified as Rutherford category 6 were excluded from the study. At our institution, clinical protocols dictate that patients presenting with extensive, irreversible tissue loss (Rutherford 6), where limb salvage is deemed anatomically or clinically futile, are managed primarily via primary major amputation or palliative care rather than undergoing attempts at arterial revascularization. Therefore, they did not meet the operative framework of this study.

#### 2.2.2. Assessment of Sarcopenia Markers

All CT scans were acquired using the same multidetector scanner model. Measurements were performed preoperatively on contrast-enhanced computed tomography angiography (CTA) at the mid-L3 vertebral level using standardized axial slices obtained in the arterial phase. On a representative axial image, both psoas muscles were manually delineated using dedicated imaging software (OsiriX MD, Pixmeo SARL, Geneva, Switzerland; version 14.1.0). For each patient, mean cross-sectional area (PMA, cm^2^) and mean muscle density (PMD, Hounsfield units) were recorded. LPMA was calculated as the product of PMA and PMD. All measurements were performed by a single trained observer. Previous validation within our group demonstrated excellent inter-observer reproducibility for psoas-derived parameters [7].

#### 2.2.3. Procedural Case-Mix

Aorto-iliac interventions consisted predominantly of endovascular angioplasty and stenting of the common and external iliac arteries using balloon-expandable or self-expanding bare-metal stents, drug-eluting stents, and covered stents. Open reconstructions included aorto-bifemoral and axillo-bifemoral bypasses with prosthetic grafts, frequently combined with common femoral endarterectomy in hybrid procedures (iliac stenting plus femoral endarterectomy). In the femoropopliteal subgroup (*n* = 77), 47% of procedures were endovascular, 10% hybrid, and 43% open reconstructions (Table S3). Infrapopliteal interventions (*n* = 14) were exclusively endovascular and primarily consisted of plain balloon angioplasty of tibial vessels. Drug-coated balloons were not routinely used, and no tibial stents were implanted. Multivessel tibial recanalizations were common.

In selected cases, minor amputations (toe or limited forefoot procedures) were performed concomitantly. Major amputations (below-knee) were reserved for patients with extensive tissue loss or uncontrolled infection. No hybrid or open reconstructions were performed in the isolated infrapopliteal subgroup. In the multilevel disease subgroup (*n* = 76), procedures typically addressed both inflow (aorto-iliac) and outflow (femoropopliteal or infrapopliteal) segments using endovascular, hybrid, or open techniques.

### 2.3. Statistical Analysis

Continuous variables were expressed as mean and median with interquartile range (IQR). Categorical variables were presented as counts and percentages. Associations between categorical variables were assessed using the χ^2^ test or Fisher’s exact test, with statistical significance set at *p* < 0.05.

The primary objective of the regression modelling was to explore the predictive value of imaging-derived muscle parameters for the length of hospital stay. To minimise the impact of highly influential observations, we first fitted an initial linear model with log(LOS) as the outcome and screened influential observations using Cook’s distance (cutoff 4/*n*). The model based on this filtered dataset (excluding observations exceeding the threshold) was predefined as our primary analysis. Conversely, the analysis encompassing the full, unfiltered cohort was performed and presented within the Supplementary Material as a supportive sensitivity analysis to confirm the robustness and stability of our estimates. Multicollinearity among predictors was evaluated using the variance inflation factor (VIF), and predictors with VIF values greater than 10 were excluded from the models. Candidate predictors included: procedure type, age, sex, comorbidities (hypertension, diabetes, chronic kidney disease, dialysis therapy, history of myocardial infarction, neurological disease), current/former smoker status, previous vascular interventions, and one imaging-derived muscle parameter (PMA, PMD or LPMA). Bidirectional stepwise selection based on the Akaike Information Criterion (AIC) was applied to identify the optimal subset of predictors for each key parameter. The predictors retained after this selection process were used in the final regression models. Results from the linear regression models were reported as β coefficients with standard errors, t statistics, *p* values, exponentiated β, and relative percentage change in length of stay (LOS).

Because LOS showed a right-skewed distribution, we used multivariable linear regression models on log-transformed LOS. Coefficients are presented as β with standard errors, t and *p* values; exp(β) represents the multiplicative effect on LOS (i.e., relative percent change). Influential observations were screened using Cook’s distance (threshold 4/*n*) and excluded if above this cutoff. Residual diagnostics included Q–Q plots for normality, residuals versus fitted values for homoscedasticity, Breusch–Pagan tests for heteroscedasticity, and inspection of influence metrics.

All analyses were conducted in R (version 4.1) using the stats [8] package for regression fitting, Cook’s distance, χ^2^, Fisher’s exact test; MASS [9] for AIC-based stepwise selection; car [10] for multicollinearity assessment; and ggplot2 [11] for all graphical outputs. Additionally, to address procedural heterogeneity, we performed stratified multivariable linear regression analyses within each procedural subgroup (endovascular, hybrid, and open surgery). Within each stratum, the same modelling strategy was applied, including assessment of multicollinearity, bidirectional AIC-based variable selection, and evaluation of model assumptions. Imaging-derived muscle parameters were entered separately into the models to avoid collinearity between related measures.

To evaluate the impact of body-size normalization, all stratified analyses were repeated using the psoas muscle index (PMI), calculated as muscle area divided by height squared (cm^2^/m^2^), in accordance with established sarcopenia literature. Effect estimates were compared between raw LPMA and normalized PMI models. For interpretability, clinically meaningful contrasts were additionally calculated by estimating the relative change in LOS between the 25th and 75th percentiles of LPMA and PMI within each procedural subgroup. These estimates were derived from the exponentiated regression coefficients of the log-transformed models. No predefined cut-off values were applied, and muscle parameters were analysed as continuous variables to preserve statistical power and avoid information loss associated with arbitrary dichotomization.

## 3. Results

### 3.1. Baseline Characteristics

A total of 234 patients undergoing lower limb revascularization for CLTI (Rutherford categories 4–5) were included. Both aortoiliac and infrainguinal disease patterns were represented. The median age was 68 years (IQR 64–74.8), and 153 patients (65.4%) were male. Median height was 170 cm (IQR 164–176), median body weight 72 kg (IQR 62–84.5), and median BMI 25 kg/m^2^ (IQR 23–29).

The prevalence of comorbidities was substantial: coronary artery disease was present in 61 patients (26.1%), prior myocardial infarction in 38 (16.2%), hypertension in 151 (64.5%), diabetes mellitus in 101 (43.2%), heart failure in 19 (8.1%), and COPD in 12 (5.1%). Chronic kidney disease stage ≥4 was observed in 19 patients (8.2%), including 7 (3.0%) on chronic dialysis. A history of prior peripheral vascular interventions was recorded in 85 patients (36.5%), and prior stroke or transient ischemic attack in 29 (12.4%). Active or former smoking was reported by 166 patients (70.9%).

Most admissions were urgent (179 patients, 76.5%). The median length of hospital stay for the entire cohort was 6.0 days. Mean PMA was 11.20 cm^2^, mean PMD 38.18 HU, and mean LPMA 432.63 cm^2^·HU (Table 1). Smokers were younger and more frequently underwent urgent or endovascular procedures (Table S5).

### 3.2. Regression Analysis

In the log-transformed LOS model, exp(β) represents the multiplicative change in hospital stay duration. For example, exp(0.69) = 1.99 indicates that hybrid procedures were associated with a ~99% longer LOS compared with endovascular treatment.

Procedure type was the strongest determinant of LOS. Compared with endovascular treatment, hybrid and open procedures were associated with approximately 100% (β = 0.69; *p* < 0.001) and 108% (β = 0.73; *p* < 0.001) longer hospitalization, respectively.

Higher LPMA was independently associated with shorter LOS (β = −0.00049; *p* = 0.004), corresponding to a 0.05% reduction per unit increase. The current/former smoker status was associated with shorter LOS (−19%; *p* = 0.003), while dialysis dependence showed a non-significant trend toward longer hospitalization (+43%; *p* = 0.056). A history of myocardial infarction was associated with shorter LOS (−18%; *p* = 0.030). Hypertension and prior peripheral interventions were not significant predictors (Table 2).

To enhance clinical interpretability, LPMA effects were also expressed per standard deviation (SD) and per 100 cm^2^·HU increment. One SD (186.7 cm^2^·HU) was associated with an 8.7% shorter LOS (exp(β) = 0.913; 95% CI 0.860–0.971). Per 100 cm^2^·HU increase, LOS decreased by approximately 4.7% (exp(β) = 0.953; 95% CI 0.922–0.984).

In comparative model analyses, LPMA demonstrated the best overall fit among sarcopenia parameters (adjusted R^2^ = 0.281; AIC = 402), outperforming PMA and PMD. Results were consistent after exclusion of influential observations (Supplementary Tables S7 and S8).

In a complementary logistic model using prolonged LOS (≥75th percentile) as the outcome, the LPMA association remained directionally consistent but was no longer statistically significant (*p* = 0.595). While this loss of significance does not invalidate our primary findings, it should be interpreted with appropriate caution, as it suggests that the predictive value of LPMA may be sensitive to outcome modeling and the statistical power limitations inherent to data dichotomization. Consequently, no definitive clinically meaningful LPMA cut-off was proposed based on this binary framework.

Multicollinearity was negligible, and LASSO regression confirmed coefficient stability, with procedure type remaining the dominant predictor and LPMA retaining a similar effect size and direction across models.

## 4. Discussion

Chronic limb-threatening ischemia (CLTI) represents the most advanced stage of peripheral arterial disease and is associated with substantial risks of limb loss, cardiovascular events, and mortality [12,13]. As the population ages and cardiometabolic risk factors persist, the burden of CLTI is expected to increase [13,14]. Patients undergoing revascularization are often elderly, multimorbid, and physiologically vulnerable. In this context, prolonged hospitalization is not a neutral outcome but is associated with increased risk of complications, functional decline, and resource utilization [15,16,17,18,19,20,21]. This physiological vulnerability is also well documented in other fields of vascular surgery. In patients undergoing complex thoraco-abdominal aortic repairs or elective endovascular aneurysm repair (EVAR), objective markers of frailty and CT-defined sarcopenia consistently predict poor outcomes and longer recovery [22,23]. Linking CLTI to this broader research shows that markers of physiological reserve, including LPMA, share a common relevance across different vascular procedures. They could function as practical clinical indicators for risk assessment before major surgery.

In this study, procedure type emerged as the dominant determinant of length of stay, with hybrid and open reconstructions associated with approximately twofold longer hospitalization compared with endovascular treatment. This finding underscores the major influence of procedural invasiveness on early postoperative recovery. Beyond procedural factors, lower LPMA was independently associated with longer LOS after adjustment for procedure type and comorbidities. Although the magnitude of this association was modest compared with procedural effects, it remained consistent across models and sensitivity analyses. Clinical recovery remains a multifactorial process where procedural complexity, baseline CLTI severity, local wound burden, and the occurrence of perioperative complications dictate the length of hospital stay. Consequently, this modest relationship must not imply that LPMA alone can meaningfully drive hospitalization duration in isolation. Instead, our findings support the concept that imaging-derived muscle parameters reflect aspects of physiological reserve that are not fully captured by traditional clinical risk factors, serving as a useful supportive marker within a comprehensive clinical assessment.

LPMA integrates muscle quantity and density and can be derived from routinely acquired preoperative CT scans without additional cost or patient burden. In contrast to subjective frailty or nutritional scores, it provides an objective and reproducible estimate of muscle status. Similar associations between sarcopenia and adverse postoperative outcomes have been reported across multiple surgical disciplines, including colorectal, cardiac, and oncologic surgery [24,25,26].

Importantly, LPMA should be interpreted as an adjunct marker rather than a primary driver of resource utilization. While procedure type largely determines early recovery trajectories, baseline muscle reserve may influence an individual patient’s capacity to tolerate surgical stress and recover efficiently. In this context, LPMA may contribute to more nuanced preoperative risk stratification.

An unexpected finding was the association between active smoking and shorter LOS. This observation likely reflects residual confounding related to age and procedural case-mix rather than any protective effect of smoking. Similar findings, sometimes described as the “smoker’s paradox,” have been reported in cardiovascular cohorts and are typically explained by differences in baseline characteristics rather than biological benefit [27,28]. Smoking remains a major risk factor for adverse vascular outcomes.

From a clinical perspective, identification of sarcopenia before surgery may help highlight patients with reduced physiological reserve. Evidence from other surgical fields suggests that structured perioperative interventions, including early mobilization and targeted nutritional support, can improve recovery in high-risk populations [29,30,31,32,33]. However, given the retrospective design of our study and the urgent nature of many CLTI admissions, these potential clinical strategies remain hypothesis-generating in this specific patient population. Prospective, randomized validation is required to determine whether targeted prehabilitation or nutritional optimization can genuinely mitigate risk and reduce the length of hospital stay in real-world CLTI pathways.

Prolonged hospitalization itself is associated with downstream risks, including readmission and mortality [34]. By linking LPMA to LOS, our study suggests that muscle reserve may represent a modifiable pathway within the broader framework of perioperative optimization. However, our findings should be interpreted in light of the retrospective design, single-center setting, and sample size. External validation and prospective studies are needed to determine whether LPMA-guided interventions improve clinically meaningful outcomes.

In conclusion, while procedural factors remain the principal determinant of length of stay after lower limb revascularization for CLTI, CT-derived LPMA demonstrates a modest but independent association with hospitalization duration. These findings support further exploration of imaging-based muscle assessment as part of comprehensive preoperative evaluation in this high-risk population.

### Limitations

This study has several limitations. First, it was a retrospective, single-center analysis, which may limit generalizability. Although LPMA is an objective imaging marker, it captures only structural muscle characteristics and does not assess muscle function or patient-reported outcomes. Despite multivariable adjustment, unmeasured confounding factors—such as socioeconomic status, baseline nutritional status, or functional reserve—may have influenced the results. Our multivariable analysis revealed counterintuitive associations, indicating that active smoking and a history of myocardial infarction were independently associated with a shorter duration of hospitalization. These unexpected findings must be interpreted with caution, as they likely reflect residual confounding or selection bias inherent to retrospective data. Given the exploratory nature of this study, which primarily targeted the predictive performance of LPMA, a comprehensive decomposition of these secondary cardiovascular covariates was not performed and remains a limitation.

Another important limitation of this study is the potential for selection bias resulting from the exclusion of 163 patients who lacked preoperative CTA imaging capturing the L3 vertebra level. Standardized assessment of PMA and PMD strictly requires this anatomical landmark; thus, their exclusion was necessary to maintain methodological rigor and prevent measurement bias. Notably, these excluded patients exhibited a significantly shorter length of hospital stay (LOS) compared to the analyzed cohort. This variance can be attributed to the retrospective inclusion of both emergent cases—who underwent rapid, targeted interventions without comprehensive abdominal imaging—and elective patients admitted with focused, external lower-limb vascular studies. The findings are therefore most generalizable to CLTI patients managed within CTA-based pathways and may not fully reflect extremely urgent cases in which comprehensive imaging is not obtained.

The median age of our cohort (68 years) may reflect local referral patterns and could differ from population-based CLTI series, limiting direct cross-study comparability. CLTI presentation and revascularization strategies were heterogeneous across anatomical segments and procedural complexity. Although we adjusted for procedure type, and performed stratified subgroup analyses to mitigate this, residual confounding related to anatomical distribution and disease severity may persist. Furthermore, because this study is inherently exploratory and hypothesis-generating, it aimed to establish the preliminary predictive value of LPMA rather than define final clinical thresholds. Subgroup-specific analyses were limited by small sample sizes in certain strata (e.g., infrapopliteal disease), and larger multicenter cohorts are required for segment-level validation.

Additionally, a relevant clinical confounding factor is the performance of concomitant minor amputations (such as toe or limited forefoot resections) and the subsequent requirement for intensive local wound care and infection control. These interventions are frequently necessary in Rutherford category 5 patients and are well known to independently prolong hospitalization. Due to the retrospective nature of our data, we could not adjust for minor amputations as an independent covariate in the multivariable models, which remains a limitation that should be addressed in future, precisely matched prospective trials.

Moreover, the inclusion criterion allowing for a preoperative CTA up to 12 months before surgery may introduce a potential confounding factor. In CLTI patients, clinical and metabolic parameters, including muscle mass and nutritional status, can fluctuate rapidly due to the acute catabolic state and infection burden. This timeframe was utilized pragmatically to accommodate a small minority of patients with high-quality external imaging and to avoid unnecessary repeat radiation and contrast exposure. However, the retrospective nature of our database precluded a restricted sensitivity analysis within a 30- or 90-day window, which should be prioritized in future prospective trials.

Due to the retrospective design and the specific structure of the available dataset, we were unable to provide a granular stratification and sub-analysis differentiating between Rutherford category 4 (rest pain) and category 5 (minor tissue loss). Since tissue loss carries a distinct metabolic, wound-healing, and infectious burden compared to rest pain alone, the varying severity within the CLTI spectrum may independently influence the duration of hospitalization. This factor could not be adjusted for in our multivariable models and remains a limitation of the current analysis.

Finally, a meaningful limitation of this analysis is the absence of comprehensive clinical severity markers, most notably the Society for Vascular Surgery (SVS) Wound, Ischemia, and foot Infection (WIfI) staging system. Due to the retrospective design and the historical structure of our institutional database (2018–2021), granular data regarding precise wound extent, infection status, localized gangrene severity, and individual post-operative wound care requirements were not systematically captured in a structured format. While the general presence of tissue loss was broadly reflected via Rutherford category assignment, the lack of a standardized WIfI clinical grading system remains a confounding factor.

## 5. Conclusions

In patients undergoing lower limb revascularization for CLTI, lower LPMA was independently associated with longer hospital stay after adjustment for procedural and clinical factors. Although procedure type remained the principal determinant of LOS, LPMA demonstrated a modest but consistent association with recovery duration. These exploratory findings support further exploration of CT-derived muscle assessment as an adjunct, hypothesis-generating tool for preoperative risk stratification in high-risk vascular patients. Prospective, multicenter studies are needed to validate these results and to determine whether targeted perioperative strategies in patients with low muscle reserve translate into improved clinical outcomes.

## Figures and Tables

**Table 1:** Baseline characteristics of the study population.

Variable	Mean	Median	Prevalence
Male			153 (65.4%)
Age (years)	69.15	68	
Height (cm)	168.92	170	
Weight (kg)	73.60	72	
BMI	25.68	25	
CAD			61 (26.1%)
History of MI			38 (16.2%)
HA			151 (64.5%)
DM			101 (43.2%)
HF			19 (8.1%)
COPD			12 (5.1%)
Earlier peripheral interventions			85 (36.5%)
CKD			19 (8.2%)
Dialysis			7 (3.0%)
History of stroke or TIA			29 (12.4%)
Smoking			166 (70.9%)

**Table 2:** Multivariable linear regression model for log-transformed length of stay (LOS).

Term	β (Estimate)	SE	*p* Value	Exp(β)	Relative Change in LOS (%)
Intercept	1.65	0.11	<0.001	5.22	—
Hybrid procedure	0.69	0.10	<0.001	1.99	+99%
Open surgical procedure	0.73	0.07	<0.001	2.08	+108%
Mean LPMA	−0.00049	0.00017	0.004	0.9995	−4.7% per 100 cm^2^·H
Smoking	−0.21	0.07	0.003	0.81	−19%
Dialysis	0.36	0.19	0.056	1.43	+43%
History of myocardial infarction	−0.19	0.09	0.030	0.82	−18%
Hypertension	0.10	0.06	0.14	1.10	+10%
Previous peripheral intervention	0.10	0.07	0.14	1.10	+10%

β (Estimate)—regression coefficient from the linear model with log-transformed LOS; Exp(β)—multiplicative effect on LOS; relative change (%) = (Exp(β) − 1) × 100.

## Data Availability

The data is available on request.

## Supplementary Materials

The following supporting information can be downloaded at: https://www.mdpi.com/article/10.3390/diagnostics16111621/s1, Figure S1. Q–Q plot of standardized residuals from the final linear regression model (log-transformed LOS as the dependent variable). Table S1. Comparison of included vs. excluded CLTI revascularization patients. Table S2. Procedural case-mix and device classes in the aorto-iliac subgroup. Table S3. Procedural case-mix and device classes in the femoropopliteal subgroup (*n* = 77). Table S4. Procedural case-mix and device classes in the infrapopliteal (BTK) subgroup (*n* = 14). Table S5. Comparison of smokers and non-smokers in the study cohort. Table S6. Length of hospital stay according to procedure type. Table S7. Length of hospital stay according to LPMA tertiles. Table S8. The model performance table on full cohort. Table S9. The model performance table on non-influential. Table S10. Generalized variance inflation factors (GVIF) for predictors included in the stepwise AIC model. Table S11. Comparison of stepwise AIC and LASSO estimates. Table S12. Stratified multivariable models—LPMA. Table S13. Stratified multivariable models—PMI (height-normalized).

## Abbreviations

The following abbreviations are used in this manuscript:

CLTI	Chronic limb-threatening ischemia
LOS	Length of hospital stay
LPMA	Lean psoas muscle area
PMA	Psoas muscle area
PMD	Psoas muscle density
PMI	Psoas muscle index
CTA	Computed tomography angiography
