# Supplementary material for: Lean Psoas Muscle Area Is Associated with Length of Stay After Lower Limb Revascularization for CLTI
Source: Diagnostics (Basel). 2026 May 26;16(11):1621. doi: 10.3390/diagnostics16111621 (PMC13256708; doi:10.3390/diagnostics16111621)
Supplement: Supplementary file 1 [file diagnostics-16-01621-s001.zip › Table-S6.pdf]

Table S6. Length of hospital stay according to procedure type

Procedure type	n	Mean (days) ± SD	Median (IQR)
Endovascular (PTA)	127	5.4 ± 4.2	4 (3–6)
Hybrid	32	11.3 ± 14.6	7 (5.8–10.3)
Open surgery	75	8.5 ± 3.4	8 (6–10)
