# Supplementary material for: Lean Psoas Muscle Area Is Associated with Length of Stay After Lower Limb Revascularization for CLTI
Source: Diagnostics (Basel). 2026 May 26;16(11):1621. doi: 10.3390/diagnostics16111621 (PMC13256708; doi:10.3390/diagnostics16111621)
Supplement: Supplementary file 1 [file diagnostics-16-01621-s001.zip › Table-S7.pdf]

Table S7. Length of hospital stay according to LPMA tertiles

LPMA tertile	n	Mean (days) ± SD	Median (IQR)
Low	78	8.3 ± 9.7	6 (4–9.8)
Medium	78	7.2 ± 5.4	6 (4–9)
High	78	6.2 ± 3.9	6 (3–9)
