# Supplementary material for: Lean Psoas Muscle Area Is Associated with Length of Stay After Lower Limb Revascularization for CLTI
Source: Diagnostics (Basel). 2026 May 26;16(11):1621. doi: 10.3390/diagnostics16111621 (PMC13256708; doi:10.3390/diagnostics16111621)
Supplement: Supplementary file 1 [file diagnostics-16-01621-s001.zip › Table-S8.pdf]

Table S8. The model performance table on full cohort:

	n	R^2	Adjusted R^2	AIC	BIC	logLik
Mean LPMA	228	0.306	0.281	368	402	-174
