# Supplementary material for: Lean Psoas Muscle Area Is Associated with Length of Stay After Lower Limb Revascularization for CLTI
Source: Diagnostics (Basel). 2026 May 26;16(11):1621. doi: 10.3390/diagnostics16111621 (PMC13256708; doi:10.3390/diagnostics16111621)
Supplement: Supplementary file 1 [file diagnostics-16-01621-s001.zip › Table-S10.pdf]

Table S10, Generalized variance inflation factors (GVIF) for predictors included in the stepwise AIC model

	GVIF	Df	GVIF^(1/(2*Df))
Procedure type	1.1429	2	1.0340
Mean_LPMA	1.0269	1	1.0134
Smoking	1.1097	1	1.0534
Dialysis	1.0231	1	1.0115
MI	1.0461	1	1.0228
HA	1.0397	1	1.0196
Previous interventions	1.0524	1	1.0259
