# Supplementary material for: Lean Psoas Muscle Area Is Associated with Length of Stay After Lower Limb Revascularization for CLTI
Source: Diagnostics (Basel). 2026 May 26;16(11):1621. doi: 10.3390/diagnostics16111621 (PMC13256708; doi:10.3390/diagnostics16111621)
Supplement: Supplementary file 1 [file diagnostics-16-01621-s001.zip › Table-S11.pdf]

Table S11. Comparison of stepwise AIC and LASSO estimates:

Predictor	Stepwise AIC estimate	LASSO λ_min estimate
Procedure t. 1 (hybrid)	0.602	0.593
Procedure t.2 (open)	0.629	0.682
Mean_LPMA	–0.000491	–0.000366
Smoking	−0.171	-0.137
Dilaysis	0.427	0.197
MI	−0.206	-0.135
HA	0.126	0.055
Previous interventions	0.120	0.055
DM	-	0.015
HF	-	0.059
eGFR_below30	-	0.083
