# Supplementary material for: Lean Psoas Muscle Area Is Associated with Length of Stay After Lower Limb Revascularization for CLTI
Source: Diagnostics (Basel). 2026 May 26;16(11):1621. doi: 10.3390/diagnostics16111621 (PMC13256708; doi:10.3390/diagnostics16111621)
Supplement: Supplementary file 1 [file diagnostics-16-01621-s001.zip › Table-S12.pdf]

Table S12. Stratified multivariable models – LPMA
Outcome: log(LOS)

**Procedural group**	**n**	**β (LPMA)**	**p value**	**exp(β)**	**Interpretation**
Endovascular	119	−0.00103	0.0003	0.99897	Significant, shorter LOS
Hybrid	27	−0.00066	0.026	0.99934	Significant (small n)
Open surgery	69	Not retained	—	—	No independent association
