# Supplementary material for: Lean Psoas Muscle Area Is Associated with Length of Stay After Lower Limb Revascularization for CLTI
Source: Diagnostics (Basel). 2026 May 26;16(11):1621. doi: 10.3390/diagnostics16111621 (PMC13256708; doi:10.3390/diagnostics16111621)
Supplement: Supplementary file 1 [file diagnostics-16-01621-s001.zip › Table-S13.pdf]

Table S13. Stratified multivariable models – PMI (height-normalized)
Outcome: log(LOS)

**Procedural group**	**n**	**β (PMI)**	**p value**	**exp(β)**	**Interpretation**
Endovascular	119	−0.1526	0.007	0.858	Significant, shorter LOS
Hybrid	27	−0.1383	0.097	0.871	Borderline
Open surgery	69	Not retained	—	—	No independent association
