# Supplementary material for: Lean Psoas Muscle Area Is Associated with Length of Stay After Lower Limb Revascularization for CLTI
Source: Diagnostics (Basel). 2026 May 26;16(11):1621. doi: 10.3390/diagnostics16111621 (PMC13256708; doi:10.3390/diagnostics16111621)

Figure S1. *Q–Q plot of standardized residuals from the final linear regression model (log-transformed LOS as the dependent variable).*

The Q–Q plot demonstrates that the standardized residuals closely follow the theoretical normal distribution line, with only mild deviations at the extreme tails. This visual assessment supports the assumption of residual normality despite the slight departure indicated by the Shapiro–Wilk test ($W = 0.98697$, $p = 0.0473$). No pronounced curvature or systematic pattern is observed, suggesting that the log transformation of LOS adequately stabilized the residual distribution for linear modeling.

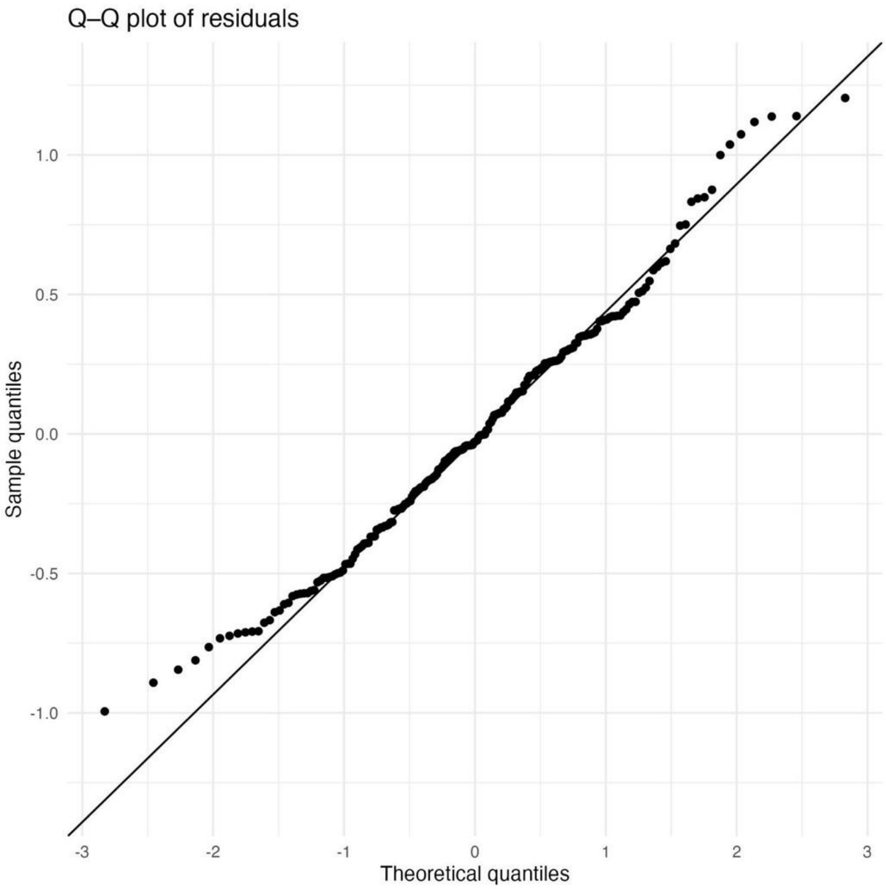

Supplement: Supplementary file 1 [file diagnostics-16-01621-s001.zip › Figure-S1.pdf]
