# Supplementary material for: Lean Psoas Muscle Area Is Associated with Length of Stay After Lower Limb Revascularization for CLTI
Source: Diagnostics (Basel). 2026 May 26;16(11):1621. doi: 10.3390/diagnostics16111621 (PMC13256708; doi:10.3390/diagnostics16111621)
Supplement: Supplementary file 1 [file diagnostics-16-01621-s001.zip › Table-S5.pdf]

Table S5. Comparison of smokers and non-smokers in the study cohort

Variable	Non-smokers (n = 68)	Smokers (n = 166)	*p*-value
Age, years (median [IQR])	73 [66–80]	66 [60–72]	<0.001
Urgent admission, %	64	78	0.04
Procedure type, %			
PTA (endovascular)	39	52	—
Hybrid	18	21	—
Open surgery	43	27	—
Comorbidities, %			
Coronary artery disease	45	31	0.03
Prior myocardial infarction	25	18	0.21
Prior PCI/CABG	16	14	0.68
Hypertension	82	74	0.19
Diabetes mellitus	47	39	0.32
Chronic heart failure	22	16	0.41
COPD	12	27	0.02
Prior peripheral intervention	9	11	0.73
CKD (eGFR < 30)	6	5	0.87
Dialysis	2	1	0.66
Neurological history	9	6	0.48
Early complications, %	24	19	0.37
