# Supplementary material for: Lean Psoas Muscle Area Is Associated with Length of Stay After Lower Limb Revascularization for CLTI
Source: Diagnostics (Basel). 2026 May 26;16(11):1621. doi: 10.3390/diagnostics16111621 (PMC13256708; doi:10.3390/diagnostics16111621)
Supplement: Supplementary file 1 [file diagnostics-16-01621-s001.zip › Table-S4.pdf]

Table S4. Procedural case-mix and device classes in the infrapopliteal (BTK) subgroup (n = 14)

Approach	n (%)	Main device / conduit	Typical target / configuration	Examples / notes
Endovascular	14 (100%)	Plain old balloon angioplasty (POBA); 2.0–3.0 mm semi-compliant or non-compliant balloons (Armada, Sterling, Advance); occasional use of long-segment (100–220 mm) balloons	Anterior tibial, posterior tibial, and peroneal arteries; occasionally dorsalis pedis	All procedures were percutaneous. Multivessel recanalizations common (≥2 tibial arteries). No stents implanted.
Hybrid	0 (0%)	–	–	Not applicable in this subgroup.
Open reconstruction	0 (0%)	–	–	No bypass or surgical reconstruction performed. Minor amputations (toe/forefoot) or wound debridement were occasionally performed concomitantly.
