# Supplementary material for: Lean Psoas Muscle Area Is Associated with Length of Stay After Lower Limb Revascularization for CLTI
Source: Diagnostics (Basel). 2026 May 26;16(11):1621. doi: 10.3390/diagnostics16111621 (PMC13256708; doi:10.3390/diagnostics16111621)
Supplement: Supplementary file 1 [file diagnostics-16-01621-s001.zip › Table-S3.pdf]

Table S3. Procedural case-mix and device classes in the femoropopliteal subgroup (n = 77)

Approach	n (%)	Main device / conduit	Typical target / configuration	Examples / notes
Endovascular	36 (47%)	Self-expanding BMS (*LifeStent, Zilver Flex, Absolute Pro*), DES (*Zilver PTX*), covered stents (*Viabahn, Tigris*), balloon angioplasty	Superficial femoral and popliteal arteries (P1–P3)	Most frequent procedure type; often multisegment re-canalization or stent relining.
Hybrid	8 (10%)	Iliac/SFA stent + CFE ± patch angioplasty	CFA–SFA junction	Commonly combined with endarterectomy and patch plasty for inflow optimization.
Open reconstruction	33 (43%)	Autologous reversed saphenous vein or prosthetic (Dacron/ePTFE 6 mm) bypass grafts	Femoropopliteal bypass above or below knee	Open reconstructions included vein (≈60%) and prosthetic (≈40%) conduits; distal targets P1–P3.
