# Supplementary material for: Lean Psoas Muscle Area Is Associated with Length of Stay After Lower Limb Revascularization for CLTI
Source: Diagnostics (Basel). 2026 May 26;16(11):1621. doi: 10.3390/diagnostics16111621 (PMC13256708; doi:10.3390/diagnostics16111621)
Supplement: Supplementary file 1 [file diagnostics-16-01621-s001.zip › Table-S2.pdf]

Table S2. Procedural case-mix and device classes in the aorto-iliac subgroup

Approach	n (%)	Main device / conduit	Typical target / configuration	Examples / notes
Endovascular	17 (63%)	Balloon-expandable BMS (*Omnilink, Formula*), self-expanding BMS (*Zilver Flex, LifeStent, Isthmus*), DES (*Zilver PTX*), covered stents (*Fluency Plus*), aortic cuff (*Zenith Renu*)	Common/external iliac arteries, distal aorta	Most procedures performed as isolated iliac stenting or re-canalization; often bilateral.
Hybrid	4 (15%)	Iliac stent + CFE/patch angioplasty (± fem-fem crossover)	Iliac–femoral junction	Combination of iliac stent implantation and open common femoral endarterectomy or crossover bypass.
Open reconstruction	6 (22%)	Woven Dacron grafts (*Gelsoft 14×7 mm, 16×8 mm, 8 mm*)	Aorto-bifemoral, axillo-bifemoral, or fem-fem crossover	Performed for extensive aorto-iliac occlusive disease or after failed endovascular repair.
