# Supplementary material for: Lean Psoas Muscle Area Is Associated with Length of Stay After Lower Limb Revascularization for CLTI
Source: Diagnostics (Basel). 2026 May 26;16(11):1621. doi: 10.3390/diagnostics16111621 (PMC13256708; doi:10.3390/diagnostics16111621)
Supplement: Supplementary file 1 [file diagnostics-16-01621-s001.zip › Table-S1.pdf]

Table S1. Comparison of included vs. excluded CLTI revascularization patients

Comparison of demographic and clinical characteristics between included (n = 234) and excluded (n = 163) CLTI revascularization patients.

p-values were calculated using t-tests for continuous variables and χ^2 tests for categorical variables.

Variable	Included (yes/no, %)	Excluded (yes/no, %)	Test type	χ^2	p-value
Age (years)	68 (median)	69 (median)	t-test	–	$p = 0.84$
BMI (kg/m²)	25 (median)	25 (median)	t-test	–	$p = 0.33$
Length of stay (days)	6 (median)	4.5 (median)	t-test	–	$p < 0.001$
Urgent admission (yes)	179 / 55 (76.5%)	65 / 98 (40.0%)	χ^2	13.81	0.0002
Smoking (yes)	166 / 68 (70.9%)	80 / 83 (49.1%)	χ^2	14.44	0.0001
Hypertension (yes)	151 / 83 (64.5%)	108 / 55 (66.3%)	χ^2	0.09	0.764
Diabetes (yes)	101 / 133 (43.2%)	72 / 91 (44.2%)	χ^2	0.03	0.865
Coronary artery disease (yes)	61 / 173 (26.1%)	49 / 114 (30.1%)	χ^2	0.79	0.374
Previous MI (yes)	38 / 196 (16.2%)	23 / 140 (14.1%)	χ^2	0.33	0.567
Previous peripheral revascularization (yes)	85 / 148 (36.5%)	67 / 96 (41.1%)	χ^2	0.89	0.344
Heart failure (yes)	19 / 215 (8.1%)	13 / 150 (8.0%)	χ^2	0.00	0.976
COPD (yes)	12 / 222 (5.1%)	10 / 153 (6.1%)	χ^2	0.19	0.662
CKD (eGFR <30)	19 / 214 (8.2%)	11 / 152 (6.7%)	χ^2	0.29	0.590
Stroke/TIA (yes)	29 / 205 (12.4%)	13 / 150 (8.0%)	χ^2	1.81	0.178
